# Medical Society of London

**Published:** 1888-03

**Authors:** 


MEDICAL SOCIETY OF LONDON.

*Nævoid Growth of Tongue.*—Mr. PITTS also showed a child with a nævoid condition of the tongue. This had gradually increased in size until it hung out of the mouth, necessitating the removal of a triangular piece of the tongue to reduce its size. It was still increasing in size, and he proposed to try the effect of multiple applications of Paquelin's cautery.

*Case of Nævus of the Tongue.*—Mr. JOHN MORGAN showed a male child, on whose tongue a nævoid growth was noticed when 18 months old. As since that time it had only increased in size *pari passu* with the growth of the tongue, and as he had on several occasions witnessed spontaneous disappearance of similar growths, he had decided to await the result of Mr. Pitts's experiment with Paquelin's cautery.

*Electric Illumination of the Male Bladder and Urethra.*—Mr. HURRY FENWICK showed a series of instruments made by Leiter of Vienna, and Hartwig of Berlin, armed with incandescent lamps for illuminating the male bladder and urethra, and demonstrated their capabilities upon patients and "dummies." He observed that endoscopy had attracted the attention and efforts of the profession since the commencement of the century, but the candle power hitherto used had proved insufficient. Quite recently (1887), however, the smallest Edison lamp had been employed, and the result had been brilliant. Instead of a cumbersome, costly, and fickle instrument, like the Nitze-Leiter endoscope of 1880, we now possessed a simple, practical, and safe apparatus by which the bladder or urethra could be examined in as strong a light as if it was viewed by direct sunlight. Every detail of the vesical or urethral surface was discernible under favorable circumstances, even to the small vessels coursing over the mucous membrane. Mr. Fenwick mentioned certain elements necessary for a successful bladder examination, and explained that the vesical endoscope needed much practice and patience before the observer could become proficient with it. He believed that the value of electric endoscopy could hardly be estimated, and he predicted that it would at once assume a high rank in the diagnosis of obscure vesico-urethral disease, and would become almost as indispensable as the opthalmoscope or laryngoscope. [Mr. Schall of Wigmore street (Mr. Leiter's agent for Great Britain) was in attendance, and showed various batteries for working the endoscopes.]

Mr. WALSHAM bore testimony to the ease with which he had been enabled to see a stone in a dummy bladder.

Dr. ROUTH asked whether the instrument had been applied to the examination of the uterus.

Mr. FENWICK, in reply, said that the instrument would doubtless be applicable to the examination of any orifice of the body.

*Case of Unilateral Sweating.*—Dr. ANDERSON showed a young man who suffered from profuse perspiration limited to the right side of the head, face, and neck. There was nothing in the man's history to give a clue as to its causation, and no history of syphilis. Two years ago, when on frontier service in Mexico, he had noticed what he called "powerlessness" of the right arm. He was unable to raise his right arm on getting up in the morning, but this generally passed off in an hour or two. The man's general health was good. He (Dr. Anderson) asked for opinions as to the cause of this curious condition and suggestions for treatment.

*Thoracoplasty.*—Mr. PEARCE GOULD read a paper on thoracoplasty, or Estlander's operation, and related the histories of four cases under his care.

CASE I. A girl, aged 9, under the care of Dr. Gilbart Smith, at the Royal Hospital for Diseases of the Chest, had in May, 1886, suffered from left empyema for two years. The left chest was considerably retracted, and there was a profuse discharge of pus from a sinus in the eighth interspace in the anterior axillary line. Mr. Gould made a vertical incision up from the sinus, and removed about an inch and a half from each of the fourth, fifth, sixth, and seventh ribs. The girl left the hospital in August, much improved in her general health, and losing only a small quantity of sero-purulent fluid from the old sinus. She was readmitted in January, 1887, as the discharge had become purulent and more abundant, and the cavity had not shown any further tendency to close. Mr. Gould repeated the former operation, and removed parts of the second, third, fourth, fifth, and sixth ribs, dividing them at the anterior and posterior limits of the empyema cavity. The child recovered and was recently shown at a meeting of the society. The cavity in the chest is completely closed, and the girl's condition is very good.

CASE II was a boy, aged 9, also under Dr. Gilbart Smith's care at the Royal Hospital for Diseases of the Chest. He was admitted in January, 1887, for fistulous empyema, from which he had suffered for two years. The left side was retracted; in it were two sinuses in the second and sixth spaces, which discharged pus freely. The boy was anæmic, thin, and very delicate looking; his urine contained one-third its volume of albumen, and his liver was considerably enlarged. The sinuses were dilated, and the cavity was carefully drained and cleansed daily. Under this treatment he improved, but after two months matters came to a standstill, and on March 28 Mr. Gould explored the chest, found a considerable cavity, and through a vertical incision removed considerable lengths of the second, third, fourth, fifth, and sixth ribs. The ribs were severed in front at their junction with their catilages, and behind at the limit of the cavity. The very thick pleura within the ribs was also freely excised. The boy recovered well, and was shown to the society. The left side of the chest is greatly flattened on all sides, and there is still a sinus which discharges a few drops daily. His general condition is excellent. The urine is free from albumen, and the liver is not to be felt below the ribs.

CASE III was a boy, aged 12, who had measles followed by pneumonia and pleurisy six years before. Paracentesis was performed twice. Further surgical aid was refused, and a year after the empyema burst externally, and had continued to discharge freely ever since, a period of five years. On admission the left chest was quite fixed, and presented three sinuses. The heart-beat was displaced outwards and upwards. On July 14 the sinuses were connected by an incision, and led into a cavity through the second space, and the tissues were removed from the second to the seventh ribs, which were then removed *in toto*, together with much thickened pleura. Hæmorrhage was considerable, but was controlled by irrigation; a counter opening was made, and the opening closed. The lad left the hospital on August 15 with the wound almost healed, and in very much improved health.

CASE IV was an adult, aged 25, who had pleurisy in 1884, which was aspirated. After a varied hospital experience, she was admitted with considerable flattening of the right chest, and with a fistulous opening at the angle of the scapula, and another in the seventh space below the nipple, which discharged abundance of fœtid pus. By means of an incision ten inches long in the axillary line, the ribs from the second to the ninth were excised, fifty-four inches in all. The pleura, which was nearly an inch thick, was also freely excised. She died suddenly the next morning. The heart was adherent throughout, and much displaced and fatty. Mr. Gould explained the object of these operations and their indications, but he deprecated resort to them merely to save time. He insisted on the necessity for exploring the cavity before operating in order to adapt the operation to each case. The success of the operation varied, but he maintained that this would be increased as it was more extensively practiced.

